# Supplementary material for: Causal Relationship between Aging and Anorexia Nervosa: A White-Matter-Microstructure-Mediated Mendelian Randomization Analysis
Source: Biomedicines. 2024 Aug 16;12(8):1874. doi: 10.3390/biomedicines12081874 (PMC11351342; doi:10.3390/biomedicines12081874)
Supplement: Supplementary file 1 [file biomedicines-12-01874-s001.zip › Supplementary Figure/Figure S8/Leaveoneout plot of MR analysis for TL to ubm-b-1720.pdf]

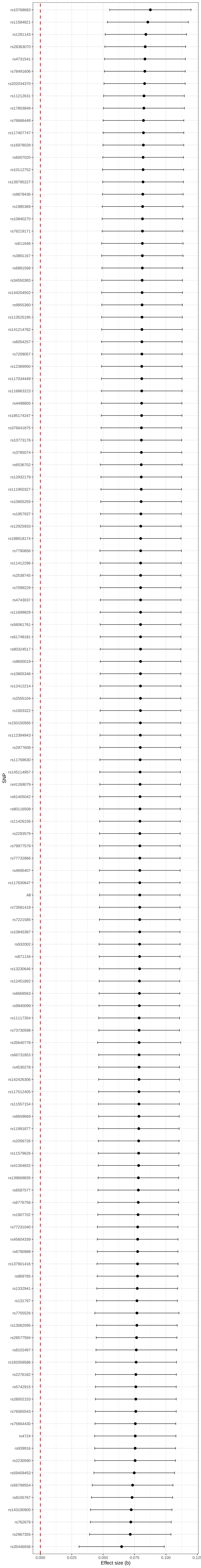
rs10768683
rs11584821
rs1291143
rs28363070
rs4731541
rs78491606
rs202034370
rs11212631
rs17803849
rs76666449
rs117407747
rs16978028
rs6007020
rs10112752
rs139795227
rs9878436
rs1985369
rs10840270
rs76219171
rs611646
rs3891167
rs6881568
rs34550383
rs144204502
rs9955360
rs113525195
rs141214782
rs6054257
rs7209057
rs12369950
rs117034449
rs116863223
rs4498805
rs185174247
rs376641875
rs10773176
rs3785074
rs6536702
rs12932179
rs111950327
rs10905255
rs1957937
rs12925933
rs188918174
rs7790856
rs11412296
rs2538745
rs7099229
rs4743037
rs11699829
rs56061761
rs61748181
rs80324517
rs9600019
rs10805346
rs12412214
rs2555104
rs1003322
rs150150565
rs112394943
rs2977608
rs11769630
rs145114957
rs41269079
rs61405042
rs80116508
rs11426156
rs2293579
rs79977579
rs77732866
rs4695407
rs117630647
A8
rs73581419
rs7221585
rs10845387
rs932002
rs871134
rs13230646
rs12451892
rs6669563
rs9940099
rs111117354
rs73730598
rs35640778
rs66731853
rs4530278
rs142426306
rs117512405
rs11557154
rs6659669
rs11991877
rs2056726
rs11579626
rs41304832
rs139669835
rs6587577
rs6776756
rs1907702
rs77231040
rs45604339
rs6790988
rs137901416
rs869785
rs1332941
rs131797
rs7705526
rs13062095
rs28577594
rs8102497
rs182059586
rs2276182
rs5742915
rs28502153
rs76065543
rs75664430
rs4724
rs939916
rs2230590
rs59409453
rs56799554
rs8105767
rs143190905
rs762679
rs2967355
rs35446936
SNP
0.000
0.025
0.050
0.075
0.100
0.125
Effect size (b)
